# Fighting Malaria in the Forests of Cambodia: A Community-Based Approach

**DOI:** 10.4269/ajtmh.19-0353

**Published:** 2020-02-05

**Authors:** Sen Tha

**Affiliations:** Malaria Focal Point at Siem Pang Health Center, Stung Treng, Cambodia

I could only access Ou Chay village by motorbike along bumpy, dust-ridden paths. Face masks and shielded helmets were essential as our team rode through clouds of dust. We planned our trip for the dry season so that we could use the motorbikes, as in the rainy season, some roads are only passable on foot. Our group comprised representatives from the Cambodian National Center for Parasitology, Entomology and Malaria Control and the WHO as well as major partners in the province, Health Poverty Action, Malaria Consortium, Catholic Relief Services, and Population Services International.

As we waited to step aboard a small raft to cross the Sekong River, we spotted a few forest rangers, suited up in their camouflage gear. These forest rangers patrol the dense forests of Virachey National Park, which surrounds Ou Chay. The forest rangers seek to prevent illegal logging, but the financial draw of logging lures villagers to risk their livelihood in the forest. Many villagers travel into the forest and stay for extended periods of time, engaging in various activities, such as wild fruit collection, hunting, slash-and-burn agriculture, and logging. While pursuing these activities, the forest goers risk exposure to malaria, especially when they are not protecting themselves in the woods with interventions such as hammock nets, repellents, and long-sleeved clothing. Understanding how to protect these forest goers was our primary objective.

Once we crossed the Sekong River, we had little to no cell phone service. Moreover, most of the forest goers do not own cell phones. How were we to find the forest goers?

Two weeks before, we had called the Ou Chay village chief to request a brainstorming session with focus group discussions about malaria in the forest. He traveled on foot and by motorbike to spread the word of our upcoming trip. We piloted our community-based approach by hosting an education session in the village’s only shop. The wooden store, situated at the side of a villager’s home, offered snacks, fresh fish, and much needed shade. As the day progressed in Ou Chay, more and more villagers gathered, some having heard the request from the chief and others seemingly just out of curiosity.

What struck me most was the hurdle of explaining why forest goers should be tested for malaria, even if they do not feel sick. Although the telltale sign of malaria is fever, there is a specific type of malaria—*Plasmodium vivax* malaria—which can remain dormant and not cause symptoms. Last year, about 70% of the total cases in Cambodia were *P. vivax*, with the majority found in forest goers.

*Plasmodium vivax* malaria can also cause relapses in a patient. During our focus group discussion, a forest goer aired his frustration about being diagnosed with malaria multiple times. With each diagnosis, he would lose days off work and earn less money for his family. Because of its ability to remain dormant, *P. vivax* can be very difficult to treat. The country is working to operationalize a drug regimen that prevents the relapses.

As much as it was a learning session for the villagers, it was also a learning session for me. For 36 years, I have worked at the Siem Pang Health Center and have dedicated my life to fighting malaria. From studying laboratory results to coordinating net distribution to supervising village malaria workers, I have served to protect the Khmer people against the disease. Although my country has achieved tremendous success during this time, we still have pockets of malaria in very remote areas, such as here in Ou Chay. I was left thinking, “How do I better communicate the importance of screening to forest goers? How do I better initiate behavioral change in remote, hard-to-reach areas such as Ou Chay?”

I learned that a crucial step to better communication is engagement of the village chief. The chief knows who frequents the forests and how best to encourage forest goers to gather in one place. I also learned that forest goers’ wives are eager to understand how screening their husbands helps protect themselves and others. Their active participation suggests that wives may be an asset for supporting behavioral change.

For the 2019 World Malaria Day, the theme was “Zero malaria starts with me.” The theme is an important reminder that protection against malaria starts with the people. As my country implements an intensified response plan to malaria, we aim to reach every Cambodian at risk through a network of mobile malaria workers. I am hopeful that a community-based approach similar to the one in a small shop in Ou Chay will support the work of mobile malaria workers and help us finally eliminate malaria.

